# Collection and Analysis of Repeated Speech Samples: Methodological Framework and Example Protocol

**DOI:** 10.2196/69431

**Published:** 2025-07-22

**Authors:** Nicholas Cummins, Lauren Louise White, Zahia Rahman, Catriona Lucas, Tian Pan, Ewan Carr, Faith Matcham, Johnny Downs, Richard Dobson, Thomas F Quatieri, Judith Dineley

**Affiliations:** 1 Department of Biostatistics & Health Informatics Institute of Psychiatry, Psychology and Neuroscience King's College London London United Kingdom; 2 School of Psychology University of Sussex Brighton United Kingdom; 3 CAMHS Digital Lab, King’s Maudsley Partnership, Department of Child & Adolescent Psychiatry Institute of Psychiatry, Psychology and Neuroscience King's College London London United Kingdom; 4 Human Health and Performance Systems MIT Lincoln Laboratory Lexington United States

**Keywords:** speech, voice, replication, longitudinal, repeat recordings, within-speaker variability, health assessment

## Abstract

**Background:**

Speech and language biomarkers have the potential to provide regular, objective assessments of symptom severity in several neurological and mental health conditions, both in the clinic and remotely. However, speech and language characteristics within an individual are influenced by multiple variables that can make findings highly dependent on the chosen methodology and study cohort. These characteristics are often not reported adequately in studies investigating speech-based health assessment, which (1) hinders the progress of methodological speech research, (2) prevents replication, and (3) makes the definitive identification of robust biomarkers problematic.

**Objective:**

This study aims (1) to facilitate replicable speech research by presenting a transparent speech collection and feature extraction protocol and design checklist for other researchers to adapt and design for their own experiments and (2) to demonstrate in a pilot study the feasibility of implementing our example in-laboratory protocol that reduces multiple potential confounding factors in repeated recordings of healthy speech.

**Methods:**

We developed a collection and feature extraction protocol based on a thematic literature review to enable a controlled investigation of within-individual speech variability in healthy individuals. Our protocol comprises the elicitation of read speech, held vowels, and a picture description and extraction of 14 example features relevant to health. We collected speech using a freestanding condenser microphone, 3 smartphones, and a headset to enable a sensitivity analysis across different recording devices.

**Results:**

We collected healthy speech data from 28 individuals 3 times in 1 day (the “day” cohort), with the same schedule repeated 8 to 11 weeks later, and from 25 individuals on 3 days within 1 week at fixed times (the “week” cohort). Participant characteristics collected included sex, age, native language, and voice use habits. Before each recording, we collected information on recent voice use, food and drink intake, and emotional state. Recording times were also documented. Analysis relating to exploring within-individual variability within the day and week cohorts, as well as the device-type sensitivity analysis, is ongoing, with findings expected later in 2025.

**Conclusions:**

The wide variability in speech data collection, processing, analysis, and reporting in research on speech’s use in clinical trials and practice is the motivation for this paper and the development of the speech curation protocol design checklist. Increased, more consistent reporting and justification of study protocols is urgently required to facilitate speech research replication and translation into clinical practice.

**International Registered Report Identifier (IRRID):**

DERR1-10.2196/69431

## Introduction

### Speech as a Digital Marker of Health

The linguistic and paralinguistic content of our speech contains rich information about our cognitive, neuromuscular, and respiratory functioning. There is a growing body of literature highlighting the potential of speech as an objective marker for disease diagnosis, monitoring, and prediction in a variety of clinical cohorts, including amyotrophic lateral sclerosis (ALS) [1], Parkinson disease [2-4], psychosis [5,6], and major depressive disorder [7,8] as has been summarized in several reviews [9-11]. Key advantages of recording speech for clinical applications include its noninvasive nature and the ability to conduct recordings both in the clinic and remotely using off-the-shelf consumer-grade audio equipment.

### Challenges and Confounders in Speech Biomarker Research

Though speech has great potential as a signal, accurately detecting changes in speech driven by changes in health is challenging, and speech markers are yet to be used as an outcome measure in clinical trials or translated for clinical use. This is partly because speech is a multifaceted, complex, dynamic signal. Many speech changes associated with different health states can be subtle, forming one part of a measured signal that is also dictated by other speaker-specific factors and recording and analysis choices. There is a pressing need to understand, quantify, and adjust for the effect of such variables, as they can mask or even mimic the effect of health changes.

Potential confounding factors related to the speaker include hormonal variations within the menstrual cycle [12,13], fatigue [14], voice use habits [15-17], emotion [18], and hydration [19]. Systematic changes with age, menopause, and medication use have also been reported [20-23]. A growing body of literature highlights the impact of methodological choices, including recording environment, hardware choices, digitization formats, and choice of extraction tools, on speech characteristics and subsequent health state analysis [24-29].

Speech elicitation strategies are another important factor in speech-based health assessment. Common strategies include (1) structured tasks, such as reading passages and vocal function exercises such as sustained phonation [30,31]; (2) semistructured tasks, such as image description exercises [32,33]; and (3) minimally structured tasks and conversational speech that, for clinical cohorts, include clinical interviews [10,11]. Choosing the right tasks is vital for ensuring the clinical validity and sensitivity of the extracted speech measures [34]; each task can produce distinct acoustic, linguistic, and emotional content and be used for the targeted capture of different aspects of speech production [34,35].

Less-structured tasks pose technical challenges, such as the need for accurate speech-to-text conversion and diarization to determine who was speaking what and when [30]. Practice effects represent another potential confounder, where recorded speech changes due to repeated exposure to a task or activities [36,37]. Although expected in speech research, practice effects are rarely documented [9,38].

Despite an awareness of such effects, methodological details and important speaker characteristics in speech-based health assessment research are underreported in the literature. These factors can be unaccounted sources of variation that become particularly pertinent when effect sizes are small or context dependent [39], which is often the case in health analyses of speech. This is of particular concern for remote data collection outside of laboratory settings where there are more degrees of freedom, for example, recording devices and geometry and the acoustic environment. This weakens replicability and hinders the development of robust methodology and tools; the discovery and verification of biomarkers; and, ultimately, clinical translation.

### Enhancing Speech Biomarker Research Through Methodology and Reporting

The lack of established methods for data collection and reporting exacerbates these issues highlighted above [40]. The Consensus Auditory-Perceptual Evaluation of Voice (Cape-V) protocol [41] and recommendations made by the American Speech-Language-Hearing Association Panel [42] are helpful starting points. However, they have limited applicability in detecting subtle changes and the broader range of speech characteristics associated with, for example, mental health and neurological disorders recorded remotely and longitudinally. These recommendations were also developed specifically for in-laboratory speech pathology assessments.

The Voiceome Study represents an attempt at standardization of longitudinal data collection for speech and language biomarker research [43]. A key feature is its recommendation of 12 speech elicitation tasks, and the study highlights that these tasks produce distinct feature clusters. However, the authors do not describe the clinical relevance of these prompts or provide any evidence base justifying their inclusion. The implications for participant burden and associated protocol acceptance and adherence by participants are also not discussed, which is an important issue in data collection [44]. The effects of the recording environment, recording time, hardware choices, and speech processing methods on the quality of extracted data are also not considered.

In conclusion, the effect of speaker-related factors and methodological choices necessitates increased reporting and justification of methods used in speech and language biomarker research, including more well-considered protocol design. To begin addressing this, we report on our detailed study protocol for collecting in-laboratory repeated speech samples from healthy individuals. Our aim in publishing this protocol is to promote transparency and reproducibility as a key step toward increased research replication and more reliable identification and validation of speech biomarkers in this domain [40,45,46]. This protocol is also a resource to replicate and adapt, including by researchers with less experience and specialist knowledge of speech processing.

## Methods

### Overview

Our pilot study builds on the literature to present an example protocol for speech corpora curation that reports and justifies methodological aspects for adaptation for other studies. We conducted this study with a specific research focus of assessing natural variability in individual voices and understanding the sensitivity of different recording devices to these variations. We collected and analyzed speech samples from healthy participants to avoid variability driven by pathology. Datasets of healthy individuals are also beneficial as baselines for comparison with clinical populations [34,47].

The consideration of participant burden and the acceptability of the protocol to participants was a part of the research process, as these factors have important implications for recruitment and protocol adherence and therefore data quality and completeness. While our protocol was designed with a specific scientific goal in mind, the core methodological aspects are relevant for researchers collecting longitudinal data to investigate other questions in speech and language biomarker research. Such research will benefit from the minimization of variations in speech between recordings due to methodological factors and clear reporting of methodology. By presenting our methodological choices in this study, we would like to enable other researchers to adapt our protocol in their own work in healthy and clinical cohorts, thereby facilitating replicable speech research.

### Protocols for Investigating Within-Individual Speech Variability

Most protocols in the literature have been a part of studies assessing localized vocal tract pathology and dysphonia, analyzing a small number of speech characteristics relevant to localized speech pathology, typically with modest-sized cohorts. Many of these studies were also conducted before remote recording and mobile devices were a consideration [48,49].

Most recently, motivated toward speech pathology vocal tract assessment, Pierce et al [50] assessed variability in repeated speech samples from healthy female participants in a remote recording study. Participants completed 1 supervised baseline recording and then recorded themselves 3 times each day for a week within prescribed intervals in a well-described protocol. The 45 participants read aloud 2 passages of text and produced sustained vowel phonations each time using a cardioid head-mounted microphone. Participants were advised to record in a quiet room with no tiling; however, adherence to this was not reported. The authors [50] analyzed 32 speech features, observing significant voice production changes over a day but no significant changes across the week. They speculate that the “worse” voice they observed in the morning could be due to (1) voice production systems affected by physiological changes due to prolonged reclining while asleep and (2) low voice use before the participants completed their first recording. Other studies have demonstrated variations in voice based on the level of hydration in participants [19,50].

Several studies motivated by mental and neurological disorder assessment have quantified within-speaker change, framed as test-retest reliability assessment. Feng et al [51] recorded 40 healthy young adults twice, 2 to 3 days apart, in the same test room, completing 7 elicitation tasks in Mandarin. They observed that only half of the 56 speech features tested had moderate test-retest reliability, as estimated using intraclass correlation (ICC). Barnett et al [52] retrospectively analyzed speech features of 46 healthy individuals recorded twice, months apart, reading aloud a “Bamboo” passage. They also observed only moderate test-retest reliability in half of the analyzed features. Stegmann et al [53] reported an analysis of 22 healthy individuals recorded daily for 7 days and clinical cohorts with ALS (72 participants) and frontotemporal dementia (24 participants) recorded approximately a week apart. They reported that the test-retest reliability, also estimated using an ICC of commonly used speech features, was well below acceptable limits for clinical use.

Each of these analyses highlights that we should expect some degree of variation in voice between repeated recordings of an individual. However, in each of these analyses, various potentially confounding methodological details such as consistency in recording time and acoustic conditions—and adherence to instructions in the unsupervised (“in-the-wild”) recordings—are not reported [12-29]. Therefore, at least in principle, measurement factors may be responsible for a proportion of the observed differences between repeated recordings of a given participant. An additional potential limitation of these works is the use of the same elicitation scripts in each recording. Increased speaker familiarity with the readings can result in practice effects [36,37], which could confound the assessment of within-individual speech variability [9,38]. Finally, to the best of the authors’ knowledge, none of the aforementioned studies have provided data (either raw audio or extracted features).

To address our chosen research question of within-individual speech variability, our protocol improves on these previous works in that we collected data at set documented times in a controlled, supervised environment and used multiple microphone types (Table 1). We also combined several structured and semistructured tasks to elicit both naive and practiced speech, with both spontaneous speech and scripted, fixed content to control for various factors. As a step toward our methodological goal of improving the reporting of methods in speech corpora curation, we present our protocol in detail in the following section.

**Table 1:** Comparison of key methodological choices in protocols of studies observing within- and between-speaker variability.

Study	Sample, n	Cohort type	Schedule	Laboratory versus remote	Microphone	Speech type
This study	28	Healthy	3/d, twice in 8-11 wk Fixed times	Laboratory	Condenser 3 phones 1 headset	R^a^, SV^b^, and PD^c^
This study	26	Healthy	3 in 1 wk Fixed days fixed time	Laboratory	Condenser 3 phones 1 headset	R, SV, and PD
Garrett and Healey [48], 1987	20	Healthy	3 in 1 d	Laboratory	Miniature condenser	R
Leong et al [49], 2013	18	Healthy	10 in 30 d, fixed time interval	Laboratory	Moving coil	R and SV
Pierce et al [50], 2021	45	Healthy	3/d in 1 wk	Remote	Headset condenser	R and SV
Barnett et al [52], 2020	46	Healthy	2 in 3-6 mo	NES^d^	NES	R
Stegmann et al [53], 2020	72	ALS^e^	Daily 1 wk	Remote	NES	R and SV
Stegmann et al [53], 2020	22	Healthy	Daily 1 wk	Remote	NES	R and SV
Stegmann et al [53], 2020	24	ALS and dementia	2 in approximately 1 wk	NES	NES	R, SV, and PD
Feng et al [51], 2024	40	Healthy	2 in 2-3 d	Laboratory	Condenser	R, SV, CS^f^, RS^g^, and DDK^h^

^a^R: read, scripted speech.

^b^SV: sustained vowels.

^c^PD: picture description.

^d^NES: not explicitly stated by authors.

^e^ALS: amyotrophic lateral sclerosis.

^f^CS: connected speech.

^g^RS: repetition of heard speech.

^h^DDK: diadokinetic rate test.

### Protocol

#### Overview

Herein, we describe our protocol, the methodological goal of which was to capture repeated speech samples with minimized measurement variability. We describe multiple methodological details relevant to wider speech and language biomarker research. To facilitate adaptation to new protocols addressing other research questions, we provide a checklist of key considerations (Multimedia Appendix 1).

This protocol’s primary scientific focus was to assess within-speaker nonpathological variation in speech over time. In the “day” cohort, we aimed to record healthy volunteers speaking (1) in the morning, afternoon, and early evening of a single day (day 1) and (2) repeatedly at the same times on a second day 8 to 11 weeks later (day 2). In the “week” cohort, our aim was to record healthy volunteers on 3 days in 1 week at the same time each day.

#### Recruitment

As a pilot study in which we sought to develop methodology, we chose to investigate variability in healthy individuals to avoid the additional variability introduced by pathology. Healthy cohorts are also valuable to establish baselines with which to compare pathological speech [34,47]. We recruited adult staff and students at the study institute, and local residents were recruited via advertisements in a research recruitment circular, institute email lists, social media, and physical flyers and posters. Potential participants were asked to read a web-based information sheet and complete a pre-enrollment screening form that repeated the eligibility criteria and collected contact details and sociodemographic data to facilitate the recruitment of a balanced cohort.

We excluded individuals aged <16 or >65 years; those aged >65 years were excluded to minimize speech effects associated with aging [23]. We also excluded smokers; those with dyslexia; and individuals currently receiving treatment for any speech, auditory, mental, neurological, respiratory, or other health disorder that could impact their speech. In addition, we excluded nonnative English speakers unless they had a sufficient level of English proficiency to read an intermediate or advanced text aloud, selecting level B2 of the Common European Framework of Reference for Languages as a threshold [54]. This was a compromise to ensure recruitment feasibility in a population with a considerable proportion of nonnative speakers in a strict timeline set by funder requirements, while minimizing confounders due to lack of reading and speaking proficiency for the specific speech elicitation tasks we were implementing.

Inclusion and exclusion criteria were provided on the web to all individuals who considered participation.

We regularly checked the cohort balance throughout recruitment to enable timely, targeted recruitment as needed. Sociodemographic groups that were underrepresented at pre-enrollment—male participants and participants aged >30 years—were prioritized for follow-up and recruitment. After an initial round of advertising, in subsequent advertising, we advertised for male participants exclusively.

Researchers emailed individuals to allocate them to 3 recording sessions in 1 day (day cohort) or week (week cohort) according to their availability and preference. Emails at each stage of participation used text templates individually adapted for more personable communication to encourage engagement. Each provisional participant’s recording sessions were scheduled, and they were emailed links to an electronic enrollment and consent form hosted on Qualtrics (Qualtrics International Inc) within 72 hours of the first session. This was to minimize the unnecessary collection of data from individuals who agreed to attend but subsequently decided not to participate.

#### Data Collection Schedule

Participants in the week cohort were scheduled for recording on a Monday, Wednesday, and Friday, fixed days that avoided the weekend to minimize confounders associated with different days of the week. Each participant in the week cohort was recorded at the same time on each day, to also minimize within-day variability between recordings [50]. Participants in the week cohort were given the option to have their session start between 10 AM and 12 PM or 3 PM and 5 PM. Participants in the day cohort were scheduled for recording starting between 8 AM and 10 AM, 1 PM and 3 PM, and 5 PM and 7 PM). A minimum time between sessions of 3.5 hours was maintained to maximize the likelihood of measuring differences in speech with time of day. The same participants were scheduled to return for a second day of recording at least 8 weeks later. Day 2 of recording was scheduled for the same day of the week as day 1 and scheduled at the same times.

#### Recording Session Procedure

At each participant’s first session, researchers explained the recording procedure, and those who had not already done so before the session completed their enrollment and consent. The forms collected basic sociodemographic data, height (as a proxy of larynx length), information on the participants’ voice use habits in the previous 3 months, and their level of English, for nonnative speakers.

Before beginning the study, the project team discussed the clearest and most consistent way to instruct participants. Our aim was to make participants feel as comfortable as possible and encourage natural speech and reproducible positioning during recording. The team had regular discussions as data collection progressed on any difficulties in this regard and ways to improve participant instruction.

At the start of every recording session, participants were also asked to complete a prerecording questionnaire on Qualtrics that collected information on factors that might introduce between-recording variation in their speech. These included the times at which participants woke up and got out of bed, when they last ate and drank any liquid, the extent of their voice use that day before recording, how much sleep they had the previous night, and if they were experiencing any minor health issues that could affect their voice (Multimedia Appendix 2). The prerecording questionnaire also included the Pick-A-Mood tool [55]. Participants were also offered a drink of water at the start of each session; we recorded if they took this.

**Figure 1:**
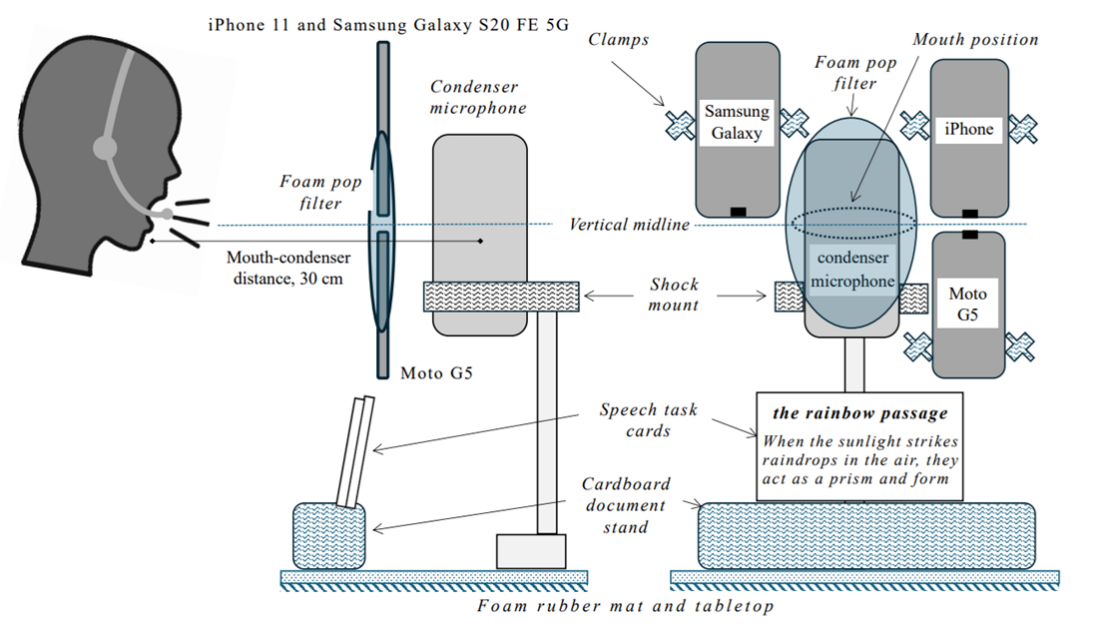
Recording setup from the side (left) and the front (right).

Participants were seated as comfortably as possible on an office chair at a desk. Their speech was recorded with an Audio Technica 2020USB+ condenser microphone on a shock mount fitted to a Rylock foam pop filter on a tabletop stand (Figure 1). The microphone was operated using Audacity open-source software running on a Dell Latitude 7440 laptop (i5 core, 16 GB RAM) running Windows 11 (Microsoft Corporation). The microphone gain was set to a fixed value at the start of every session to maximize the signal-to-noise ratio while avoiding clipping. Participants were positioned 30 cm from the condenser microphone, the distance at which the device’s frequency response is specified. The chair’s height and left-right position were adjusted so the participant’s mouth was level with the pop filter and centered on the microphone. Participants were reminded not to move their chairs during the session. The participant and setup were surrounded by acoustic-absorbing foam and textiles.

We positioned 3 smartphones (Apple iPhone 11 [released 2019], Samsung Galaxy S20 FE 5G [released 2020], and Motorola G5 [released 2017]) directly adjacent to and in the plane of the pop filter with their microphones positioned on the estimated vertical midline of the condenser microphone. These positions were fixed through all recordings and were comparable to if the participant held their phone in front of them as if in a video call [7]. Smartphone positioning was checked before each session.

Participants also wore a budget consumer office headset (Plantronics Blackwire 3220). The American Speech-Language-Hearing Association Panel recommends the use of headset microphones as the microphone-mouth distance can be fixed for the duration of a recording [42]. Our headset was operated using Audacity run on a MacBook Air (Intel Core i5, 16-GB RAM; Apple, Inc), using a gain level fixed over all participants and sessions. Participants were instructed to position the headset microphone 2 finger widths from their cheek and to one side of their mouth, using a mirror as needed. The supervising researcher checked headset microphone positioning before recording.

Before commencing the elicitation tasks, the participants were instructed to complete them at their own pace and to speak at a natural volume and pace. They were also instructed to switch their phones off or into flight mode or leave the phones outside the recording room to prevent interference with the recordings.

At the beginning and end of each recording session, as well as between each exercise, the researcher running the session played an audio tone (an alarm tone on their mobile) to prompt the participant to proceed with the next speech task and to aid the manual separation of the tasks into individual audio files following the session.

Following the completion of the speech tasks, the researcher assisted the participant in removing the headset and stopped each recording device. Participants were thanked for their time and reminded of their next recording session appointment, where applicable. At the end of each participant’s final recording session, researchers asked participants to consider completing a postparticipation questionnaire. Following their departure, the project team promptly emailed participants a link to the questionnaire and codes for shopping e-vouchers, compensating them for their time.

#### Speech Elicitation Tasks

**Figure 2:**
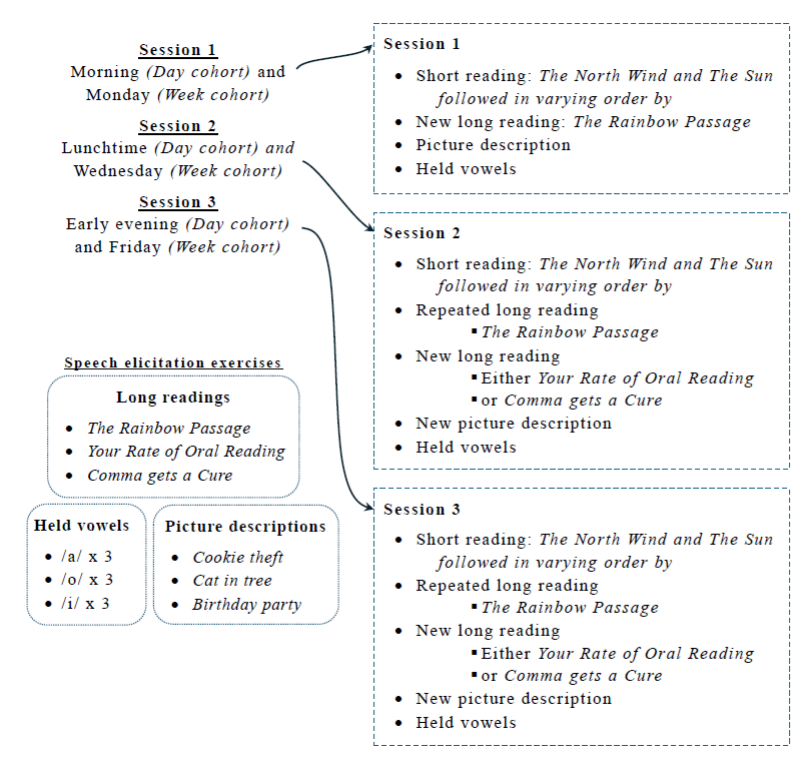
Speech elicitation overview. Our protocol elicited nonpracticed long-scripted speech in each session, plus practiced short and long readings, except in session 1. Participants described a different picture and produced held vowels in each session. The task order was varied between participants and between sessions.

Researchers provided participants with a varying combination of speech elicitation tasks in each session (Figure 2). Our choices balanced collecting several types of speech that, when combined, provide a variety of health-related indicators and sufficient amounts of each with participant burden and acceptance. A protocol with too many tasks, long recording sessions, or the elicitation of speech with personal content could deter potential participants and result in failures to complete all scheduled sessions.

Session 1 began with a short, simple reading, the “North Wind and the Sun” [56], as a form of warm-up exercise to help participants feel comfortable and settled before beginning the other tasks that would be the focus of our analyses. This was followed by a longer reading, “The Rainbow Passage” (long version) [57]; a timed picture description (up to 2 minutes) and 3 repetitions each of 3 held vowels, /a/, /o/, and /i/. In sessions 2 and 3, participants completed the 2 readings from the first session and the held vowels and an additional long reading in each, one of “Your Rate of Oral Reading” [58] and “Comma Gets a Cure” [59]. They also completed a new picture description in each of sessions 2 and 3. The elicitation task order was varied between sessions of each participant and between participants to avoid introducing systematic biases with specific tasks.

The scripted tasks provided standardized linguistic content. Repetitions of the “North Wind and the Sun” and “The Rainbow Passage” enable direct comparison of paralinguistic features for the same speech between sessions, although these repeated recordings will also be affected by practice effects. Recordings of “Your Rate of Oral Reading” and “Comma Gets a Cure” provided set linguistic content that was not subject to practice effects in the week study and in day 1 of the day study, as they were new to the participant.

We selected “Your Rate of Oral Reading” and “Comma Gets a Cure” as, along with “The Rainbow Passage,” the 3 readings have a similar lexical and linguistic complexity and length, combined with a similar phonetic balance in the literature [30,31]. Therefore, we deemed them suitable for quantifying speech variability between sessions while avoiding practice effects.

“The Rainbow Passage” and “Your Rate of Oral Reading” were selected as factual texts rather than stories to minimize the likelihood of participants using a “storytelling” voice and therefore maximize the likelihood of them speaking in their natural voices. This choice was informed by our observations in the mobile health study, Remote Assessment of Disease and Relapse—Major Depressive Disorder (RADAR-MDD) [60], where participants tended to use emphasis and be expressive in reading a story. Our choice of “Comma Gets a Cure” was a compromise; it is a story but has desirable lexical and phonetic characteristics that have been well documented in the literature [30,31].

Picture description tasks provided spontaneous speech. We used 3 images: the Cookie Theft (original version), the Cat in the Tree, and the Birthday Cake [33]. All pictures are black and white designs, depicting a simple story situation with a central focus and interacting elements. Typically used in speech assessment in neurodegenerative disorders, for example, Alzheimer disease, to investigate cognitive characteristics via the linguistic content of an individual’s speech [32], picture descriptions also have value in paralinguistic analysis [61].

Held vowel sounds provided standardized acoustic signals without any lexical, structural, or linguistic effects to account for, suitable for measurement of perturbation and quality measures [62,63]. The choice of elicitation tasks was advantageous from a data privacy perspective, as they did not elicit the disclosure of personal information.

#### Data Quality Control Checks, Storage, and Preparation

After each recording session, all audio files were named in the following format: ParticipantID_Device_Day_Session. They were then uploaded to a secure Microsoft SharePoint site maintained by King’s College London, accessible only by project staff.

The researcher running the session also completed a data quality control log that detailed (1) the start time of each session, using the timestamp on the audio files; (2) if the participant drank any water during the session; (3) any interruptions or participant behavior that could affect the recording content or quality, for example, the participant moving their chair and subsequent chair repositioning; (4) any extraneous noise during the session; (5) any issues completing the vowel task; (6) any participant difficulties completing the tasks; and (7) any other event or observation not covered by the other fields that could affect the recording.

The researcher then checked that (1) all audio files were uploaded into the correct participant and session folders, (2) each file contained recordings of the correct speaker, and (3) all tasks were completed in the stated order. The researcher also noted any additional audible issues in the data not previously captured in the quality control log.

Recordings of individual elicitation tasks were then separated into individual files using Audacity. File names were appended to include which task they contained with the following naming convention: ParticipantID_Device_Day_Session_Task.

#### Preliminary Feature Extraction

We extracted 14 example features from condenser microphone recordings of “The Rainbow Passage.” The purpose of extracting these features as part of our protocol development is to demonstrate the feasibility of our methodology pipeline. We present features captured with the condenser microphone only as our benchmark device, as example values that are not subject to any preprocessing that could erroneously affect the values extracted. These features were chosen as they are commonly used in speech-health research, representing timing and fluency characteristics and the speech production subsystems of respiration, phonation, and articulation (Table 2).

**Table 2:** Speech features extracted from the recordings to generate normative.

Features			Description
**Timing and fluency**			
	Duration, second	Length of recording	
	Speaking rate, syllables second^–1^	Total syllables divided by duration	
	Articulation rate, syllables second^–1^	Total syllables divided by total speaking time	
	Pause rate, second^–1^	Total pauses divided by duration	
**Respiration**			
	Intensity (mean), dB	Loudness of speech signal	
**Phonation**			
	Pitch (mean), Hz	Auditory perceived tone	
	Pitch (SD), semitones	SD of pitch	
	Harmonic:noise ratio (mean), dB	Extent to which harmonic structures are affected by noise	
	Spectral slope (mean)	Gradient of the voiced spectrum	
	Cepstral peak prominence (mean), dB	Amplitude of cepstral peak, relative to a regression line through the cepstrum	
**Articulatory**			
	First formant frequency (mean), Hz	First resonant frequency of the vocal tract	
	Second formant frequency (mean), Hz	Second resonant frequency of the vocal tract	
	Gravity (mean), Hz	Center frequency of the narrow band spectrum	
	Deviation (mean), Hz	Spread of frequencies around the spectral gravity	

Timing and fluency features have previously been demonstrated to contain important clinical information for conditions including depression [7,64], ALS [65], and Parkinson disease [66]. Respiration and phonation features are widely used in speech-based mental health analysis [7,9,10]. Articulation features have been included as they indicate changes in speech intelligibility and speech-motor control and have been proposed as markers for a variety of health conditions [9-11,67].

To extract these features, we first used Parselmouth [68] to convert all audio files to single-channel 16-kHz waveform audio file format files with 16-bit resolution. Our acoustic features were extracted at two levels: (1) suprasegmentally—calculated over the entire reading—and (2) for individual occurrences of open /a/vowels of at least 50 ms duration from “The Rainbow Passage.” For the /a/ vowels, we extracted the features per identified instance of the vowel and calculated the mean per recording over all instances. We provide suprasegmental acoustic features, as this is a common approach in paralinguistic analyses [69]. Extraction purely from /a/ vowels, in contrast, provides more granular, controlled acoustic measures of speech. The use of the open /a/ vowel has been recommended for more reliable extraction of voice quality measures [70]. We report results in terms of the median (IQR) for the day and week studies separately, providing a resource of normative values for use in future analysis.

As a more realistic and affordable approach toward clinical research, we implemented an automated approach to identify instances of /a/ in our files. First, we transcribed our files offline using the Open AI whisper-base.en model [71], an open-source automatic speech recognition (ASR) tool, which has been demonstrated, in independent testing, to have an average word error rate of 12.8% calculated over 9 different ASR test sets [72]. We then performed a forced alignment of the resulting transcripts using the Montreal Forced Aligner (MFA) [73] and English MFA acoustic model (version 2.0.0a). After identifying the vowels in the phonetic alignment, we extracted the features per identified vowel then took the per-participant, per-session mean of these features to form our final representation. We performed spot-checks of the accuracy of these alignments, dictated by timing and budgetary constraints. Forced alignment software is generally considered reliable, giving near human-level alignments [74,75]. We conducted cursory spot-checks to assess the reliability of alignments rather than performing a more formal analysis. Due to time and budget constraints, these checks were conducted “by ear” in Praat using the MFA-generated TextGrids to isolate the identified vowels. No specific alignment scores are provided as we lacked annotated ground truth data.

Features were extracted using Parselmouth [68], an open-source Python library that enables the use of Praat, a software package for speech analysis [76]. Speech timing features are extracted using intensity thresholds [77]. All prosodic, phonation, and articulatory measures were extracted using default Praat settings, except for the extraction of F0, which followed the 2-step approach recommended in the study by Vogel et al [64], and cepstral peak prominence, which followed settings recommended in the study by Murton et al [78].

#### Summary

Our protocol is unique (Table 1): it collects data using multiple microphone types in a controlled environment to control for and minimize variability attributable to hardware, setup, and acoustic conditions. The speech elicitation prompts enable the collection of acoustically rich and varied content while (1) containing a core amount of fixed phonetic content to enable comparable analyses and (2) introducing new readings in each session to minimize potentially confounding practice effects. We collated a list of factors we considered in designing our protocol that may be used as a framework by other researchers designing speech collection protocols (Multimedia Appendix 1).

### Ethical Considerations

The pilot study received approval from the Research Ethics Committee of King’s College London (reference LRS/DP-22/23-36194). As part of our pre-enrollment screening and again in our consent process, participants were asked to confirm their understanding of these criteria and reminded that they should not take part if they did not meet any of them. We did not collect any information in relation to these criteria for ethical reasons and as Article 5(1)(c) of the General Data Protection Regulation stipulates the collection of minimal necessary data [79]. Detailed health information was superfluous to our study aims and of a sensitive personal nature.

Upon completion of the recordings, participants were compensated for their time with e-vouchers redeemable in several shops. For the day cohort participants, these comprised £20 (US $25) for 3 sessions (day 1) and £60 (US $75) for 3 sessions (day 2) to encourage completion of both days. The participants of the week study received £40 (US $50) for 3 sessions.

## Results

**Figure 3:**
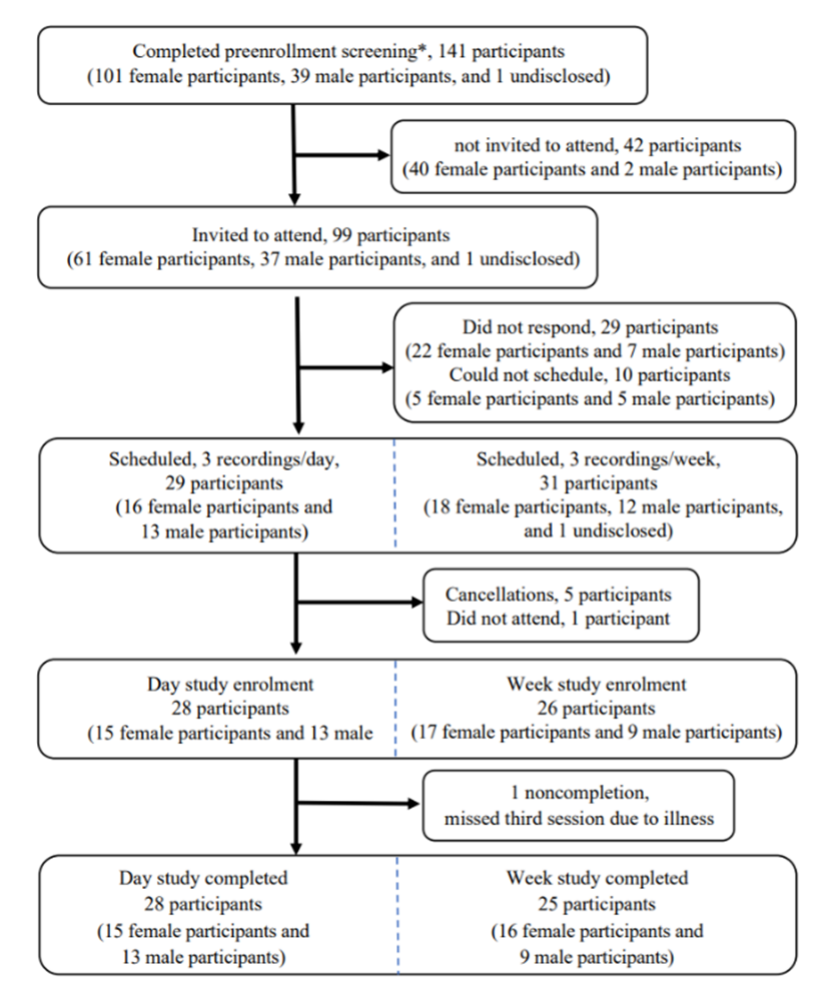
Strengthening the Reporting of Observational Studies in Epidemiology (STROBE) flowchart describing participant recruitment, enrollment, and completion. *Preenrollment was completed via Qualtrics, email, and face-to-face communication.

Data collection and preliminary analysis was funded from April 1, 2023, to March 31, 2024. Recruitment began on June 5, 2023. Pre-enrollment screening to exclude any hearing, speaking, neurological, or mental health disorders that might affect their speech was completed by 141 participants (Figure 3). In total, 28 and 26 participants enrolled in the day and week studies, respectively (Table 3). One participant in the week study completed 2 of the 3 recording sessions due to illness (Figure 3). Day 1 recordings began on June 14, 2023, and were completed on August 10, 2023. Day 2 recording began on August 9, 2023, and was completed on October 5, 2023. Week recordings commenced on June 19, 2023, and were completed on October 6, 2023. At the time of submission of this manuscript, analysis was in progress. We plan to submit of our core analysis later in 2025.

In the day study, the median recording start times for the morning sessions were 9:12 and 9:11 for days 1 and 2, respectively (Multimedia Appendices 3 and 4). The median afternoon and evening recording start times for both days were 14:05 and 18:04, respectively. In the week study, the most common recording slots were 10 AM to 11 AM and 12 PM to 1 PM, with 5 participants each (Multimedia Appendix 5). Recording times for each participant were consistent across the Monday, Wednesday, and Friday sessions, with differences in start times all <30 (median 13, range 3-22) minutes.

In total, the study comprised 245 recording sessions and produced 1225 audio files from 5 recording devices, totaling 169 GB of data. Using Audacity, we separated the readings of “The Rainbow Passage” from the condenser microphone and extracted our 14 example speech features using the methodology previously outlined. These values are provided for the day and week study participant groups (Table 4).

The focus of this paper is methodology development. Therefore, an analysis of within-individual speech variation and the ability of different devices to capture this variation is beyond the scope of this paper; it will be reported in future publications.

**Table 3:** Participant characteristics.

Characteristics			Day (n=28)		Week (n=26)
**Sex, n (%)**					
	Female	15 (54)		17 (65)	
	Male	13 (46)		9 (35)	
Age (y), median (IQR)			26 (23-34)		29 (24-34)
Height (m), median (IQR)			1.70 (1.63-1.79)		1.71 (1.63-1.78)
**Ethnicity, n (%)**					
	Asian or Asian British (Indian, Bangladeshi, and Chinese)	5 (18)		7 (27)	
	Arab	0 (0)		1 (4)	
	Black, African, Caribbean, and Black British (Caribbean)	1 (4)		1 (4)	
	White (United Kingdom and Ireland)	14 (50)		9 (35)	
	White, other	3 (11)		5 (19)	
	Mixed or multiple ethnic groups	4 (14)		1 (4)	
	Other ethnic groups	1 (4)		2 (8)	
**Language status, n (%)**					
	Native English speaker	24 (86)		17 (65)	
	Non–native English speaker^a^	4 (14)		9 (35)	
**Voice use in the 3 mo before recording, n (%)**					
	Low	1 (4)		2 (8)	
	Intermittent	7 (25)		6 (23)	
	Regular	19 (68)		15 (58)	
	High	1 (4)		3 (12)	
**Minor health issues, n (%)**					
	Allergies	2 (7)		4 (15)	
	Sinusitis	1 (4)		0 (0)	
	Acid reflux	1 (4)		1 (4)	

^a^All B2 levels or above per the Common European Framework of Reference for Languages [54].

**Table 4:** Normative median (IQR) feature values for a set of 14 example features extracted from condenser microphone recordings of “The Rainbow Passage.”^a^

Feature extraction level^b^			Week (n=26), median (IQR)		Day 1 (n=28), median (IQR)		Day 2 (n=28), median (IQR)
**Duration (s)**							
	Suprasegmentally	122 (110 to 136)		114 (104 to 126)		113 (103 to 123)	
**Speaking rate, (syllables s^–1^)**							
	Suprasegmentally	3.69 (3.40 to 3.99)		3.72 (3.53 to 4.06)		3.72 (3.49 to 4.00)	
**Articulation rate, (syllables s^–1^)**							
	Suprasegmentally	4.63 (4.32 to 4.91)		4.65 (4.47 to 4.87)		4.65 (4.48 to 4.91)	
**Pause rate (s^–1^)**							
	Suprasegmentally	0.233 (0.205 to 0.264)		0.215 (0.191 to 0.255)		0.214 (0.187 to 0.251)	
**Pitch mean (Hz)**							
	Suprasegmentally	187 (120 to 202)		146 (111 to 194)		154 (112 to 192)	
	Automatically identified /a/ vowels	183 (124 to 203)		150 (114 to 189)		157 (115 to 196)	
**Pitch SD (Hz)**							
	Suprasegmentally	2.91 (2.54 to 3.57)		2.78 (2.35 to 3.79)		2.89 (2.41 to 3.69)	
	Automatically identified /a/ vowels	0.37 (0.26 to 0.59)		0.38 (0.22 to 0.56)		0.35 (0.21 to 0.58)	
**Intensity (dB)**							
	Suprasegmentally	68.7 (67.1 to 70.1)		68.2 (66.7 to 69.8)		68.5 (66.8 to 69.9)	
	Automatically identified /a/ vowels	72.7 (70.8 to 74.5)		72.6 (70.2 to 74.6)		73.0 (70.7 to 74.7)	
**Harmonic:noise ratio (dB)**							
	Suprasegmentally	10.43 (7.28 to 12.02)		8.33 (6.39 to 9.85)		8.34 (6.69 to 9.97)	
	Automatically identified /a/ vowels	8.57 (4.62 to 10.61)		6.44 (4.38 to 7.97)		5.58 (3.72 to 8.33)	
**Spectral slope**							
	Suprasegmentally	–17.0 (–18.6 to –15.6)		–16.4 (–17.7 to –15.4)		–16.4 (–18.0 to –15.1)	
	Automatically identified /a/ vowels	–20.5 (–22.0 to –19.1)		–19.8 (–21.6 to –18.7)		–19.7 (–22.1 to –18.7)	
**Cepstral peak prominence (dB)**							
	Suprasegmentally	10.14 (9.56 to 10.74)		9.96 (9.43 to 10.69)		10.17 (9.36 to 10.77)	
	Automatically identified /a/ vowels	13.91 (12.84 to 15.03)		13.26 (11.76 to 15.03)		13.50 (11.91 to 14.82)	
**First formant (Hz)**							
	Suprasegmentally	477 (449 to 504)		482 (454 to 507)		475 (450 to 505)	
	Automatically identified /a/ vowels	648 (577 to 698)		639 (580 to 674)		628 (571 to 678)	
**Second formant (Hz×10^3^)**							
	Suprasegmentally	1.65 (1.56 to 1.72)		1.57 (1.49 to 1.64)		1.57 (1.48 to 1.63)	
	Automatically identified /a/ vowels	1.33 (1.25 to 1.43)		1.26 (1.17 to 1.36)		1.25 (1.17 to 1.37)	
**Spectral gravity (Hz)**							
	Suprasegmentally	417 (362 to 465)		453 (388 to 487)		433 (367 to 497)	
	Automatically identified /a/ vowels	613 (511 to 687)		651 (564 to 706)		621 (544 to 696)	
**Spectral deviation (Hz)**							
	Suprasegmentally	330 (286 to 389)		363 (324 to 398)		357 (320 to 393)	
	Automatically identified /a/ vowels	361 (310 to 426)		370 (342 to 414)		362 (331 to 407)	

^a^Feature definitions are provided in Table 2.

^b^Features are extracted suprasegmentally or from automatically identified /a/ vowels in readings of “The Rainbow Passage” recorded with a condenser microphone that did not apply any preprocessing.

## Discussion

### Principal Findings

We developed a protocol and checklist for study design, implementation, and reporting of repeated speech sample recording in the same individuals over time (Multimedia Appendix 1). The metadata reporting, scheduling, device choices, elicitation tasks, data storage and preparation, and feature extraction provide an adaptable template for other researchers collecting repeated speech samples.

Our specific research focus was to gain insights into speech variation over the course of a single day and week while controlling for practice effects. The protocol is unique among studies exploring within- and between-speaker variability in a nonpathological population in the variety of speech captured and the number and type of recording devices. This allows us to observe how within-individual variability is captured by mobile devices. Analysis of these aspects will be presented in future work.

The protocol also enabled us to generate a small but well-described dataset of normative values, which are underreported in the speech biomarker literature [34,47], of 14 example features commonly used in speech-health research. The insights resulting from this work provide us with a foundation for the design of future data collection and interpretation in clinical cohorts.

### Limitations and Lessons Learned

The design and implementation of this protocol provided insights that will inform the methodology of future studies.

#### Protocol Development

The design of this protocol was made challenging by the absence of suitable established collection and reporting protocols [40]. Discipline silos are a core challenge in speech-based health assessment literature that hinders protocol development and reporting. There is a lack of teams integrating clinical-facing researchers who collect data and researchers who process and analyze the data, who are typically from engineering or computer science backgrounds. This can lead to gaps in the collection and reporting of speaker factors and methodological choices that can influence the measurement of recording speech. Consistent reporting of the effects of speaker-related, recording, and processing factors is urgently required to aid the development of robust speech collection protocols and processing pipelines [10,80] and to inform the statistical design of speech studies in clinical cohorts [47].

#### Speech Elicitation Strategies

The choice of speech elicitation strategies in any protocol is a trade-off between competing factors that include the need to capture indicators of different aspects of speech production, driven by the research question [34,35]; participant burden and adherence; and strategy-dependent technical challenges in extracting features from recordings. We prioritized a combination of strategies to elicit indicators of several aspects of speech production relevant to mental, neurological, and respiratory health that were quick and easy for participants and did not require them to disclose personal information. These may not be suitable for every biomarker study and application.

#### Resource Requirement

Although participant numbers were small (n=54), the resources required to implement all steps of the protocol—recruitment, data collection, preprocessing of audio files, and feature extraction—were substantial. Data collection and audio preprocessing were particularly labor intensive. Our 245 recording sessions extended from 8 AM to 7:30 PM. We preferred to run these sessions with 2 researchers present to help minimize the likelihood of errors, although this was often not logistically feasible. Regarding preprocessing, we estimate that splitting the 1225 audio files into their individual tasks required close to 720 hours of researcher time. This highlights the need for more efficient recording and annotation techniques to recruit large, well-powered studies.

One way to increase dataset size and minimize researcher burden when implementing a similar protocol in the future could be to collect data remotely using PCs or smart devices using collection platforms such as RADAR-base [81]. Such a solution does not require researcher time to run the recording sessions, and apps can be easily designed to record different speech elicitation activities individually, saving manual segmentation time. However, remote studies are more likely to result in missing data, incorrectly completed tasks, and more variable data quality [60,82].

Participant noncompliance, particularly in clinical cohorts, is a further concern in remote studies. Pierce et al [50] reported high adherence of 92% of their healthy participants to the prescribed recording times over 7 days. Over collection intervals of up to 18 months, we observed clinical cohort completion rates of 50% (IQR 21%-74%) and 41% (IQR 13%-67%) for the scripted and free-speaking speech tasks, respectively, in RADAR-MDD, where speech was one of >10 data streams. Within the sparse longitudinal literature, the Voiceome Study is a further example, where only 21% of participants completed ≥2 recordings [43]. Therefore, there is a need to understand participant motivation and functionality concerns in mobile data collection.

#### Recruitment Balance

Before beginning recording, we aimed to recruit a 50/50 balance of sex at birth. However, we quickly learned that this required a concerted effort to achieve in the fixed time that we had to complete our work, dictated by funder requirements. In total, 101 women completed pre-enrollment forms versus 39 men, which was only achieved following specific appeals for male participants. Our final overall cohort comprised 22 men and 32 women. While not 50/50, this is more balanced than the 75/25 female/male balance of the clinical speech cohort recruited in RADAR-MDD, which was attributed to the greater reported incidence of depression in women [7,60]. We did achieve good attendance once participants enrolled, with only 1 participant of 54 missing 1 session due to illness. This highlights that participants were engaged and willing to complete the speech tasks.

#### Recruitment Feasibility in Clinical Cohorts

The recording of healthy volunteers in this study was a deliberate design choice; a better understanding of healthy speech is needed to understand changes that accompany pathology. Our choice minimizes variability due to pathology and piloted data collection procedures for future larger studies. Adaptations may be needed to accommodate data collection in clinical populations.

When implementing our protocol, we benefited from the large pool of potential “healthy” volunteers in our institution. Clinical inclusion criteria could shrink the recruitment pool, and staff and students may be more reluctant to volunteer if it requires disclosure of a diagnosed mental health disorder. Therefore, it remains to be seen if a clinical cohort, such as participants with major depressive disorder, could be recruited for the same protocol recording in a controlled environment, given the need for set recording times and days for 3 to 6 sessions.

In separate research in a clinical cohort, we have observed that the choice of speech elicitation activity is also important for participant and patient engagement in the context of future mobile speech monitoring apps [44]. Fixed, repeated tasks increase the risk of disengagement; for example, we received participant feedback in RADAR-MDD that repeating the same reading every 2 weeks for up to 2 years became tedious. Recruitment in the Voiceome Study was high, but data contribution rates were low, and the lack of engagement was not discussed [43].

#### Metadata Collection

A range of speaker-specific factors dictate changes in speech; therefore, the collection of personal data is essential in speech-health studies, as such factors may relate to selection, information, or confounding biases. The collection of such information is a balancing act of analytical goals versus (1) ethical and regulatory considerations that dictate any personal information collected should only relate to what is needed for obtaining meaningful results; (2) participant acceptance and recruitment feasibility, as studies collecting more personal and sensitive information, which may also increase the participation time, may be more challenging to recruit; and (3) logistical considerations, depending on the time and resources available to complete data collection.

We had ethical, participant acceptability, and logistical factors in mind when deciding what information to collect in our protocol (Multimedia Appendix 2). Information that we did not collect but would recommend others consider includes (1) caffeine and alcohol intake before recording [83,84], (2) medications taken [85,86], (3) menstrual cycle phase at the time of recording and whether female speakers are menopausal [12,13], and (4) participant mood using a clinically validated tool.

As this protocol was for a pilot study, we did not consider getting feedback on metadata collection through patient public involvement work. However, this should be a core consideration when using the underlying methodology in future studies.

#### Equipment Setup

Our setup had 2 limitations with implications for speech measurement precision. First, it was possible for participants to move the position of the office chair on which they were seated during recording as it had wheels and was rotatable. This was a trade-off, as with the chair’s height adjustment feature, participants could be easily centered on the microphone setup per our protocol. We mitigated this risk by observing participants during recording and making gentle reminders no to move the chair and, in rare cases, repositioning the participants. However, participant movement could not be completely excluded.

Second, there was a limit on how close participants could position their mouths from the microphone, depending on their BMI, as the condenser microphone was set back from the table edge in a fixed position for the study to minimize adjustment of the setup and fully surround it by the acoustic foam enclosure. This had the potential to result in deviations from mouth-microphone distance in our protocol. This issue could be mitigated by positioning the microphone closer to the desk edge, combined with an extension of the acoustic foam to surround the participant and microphone more fully.

In addition, early in the study, we occasionally observed small amounts of audible interference on recordings from mobile phones and, on rare occasions, phone alert tones and incoming calls. We subsequently requested that participants switch their mobile devices off, place them in flight mode, or leave them outside the recording room during sessions. We later began to set our study phones in flight mode, after occasional, new observations of interference in sessions where interference from the participants’ phones could be excluded.

#### Feature Extraction

Our choice and specification of features to report represented a considerable challenge when developing the protocol. To the best of the authors’ knowledge, there is no agreed minimal benchmark feature set in the literature for such a purpose. In addition, the perturbation and quality measures typically reported in the voice disorder literature [62,63] are limited; they do not adequately capture all the vocal effects associated with neurological and mental health conditions.

Meanwhile, predefined multivariate feature sets, such as the extended Geneva Minimalistic Acoustic Parameter Set (eGeMAPs) or the Computational Paralinguistics Challenge Set (ComParE), available in the openSMILE toolkit [87,88] were not designed for health assessments. For example, these feature sets do not contain specific timing and fluency measures, such as pause rate, a widely used feature in the ALS and depression literature [7,8,65]. A similar feature set to ours is published in the study by Larsen et al [89], but it contains jitter and shimmer measurements, which have limited utility when extracted from connected speech [70].

An additional challenge is that many commonly reported features are not uniformly defined or extracted by different extraction tools. For example, Lenain et al [90] compared vocal jitter across 3 toolboxes and only obtained weak correlations between the different implementations.

We used Praat as it is arguably more widely used in speech pathology and phonetics research. However, a weakness of Praat we observed relates to the number of settings associated with extracting each feature; finding guidance on preferred values for these settings is difficult. We also observed that default values were not ideal in certain circumstances. For example, when testing the pitch feature extraction code, we observed that the default pitch ceiling value of 500 Hz could result in false pitch readings of >300 Hz, well outside of expected ranges for this feature.

A challenge relating to extracting features over specific vowels is reliance on third-party ASR and forced alignment tools. Our choice of Whisper and the MFA was to allow us to extract normative feature values from a processing pipeline comprising standard, open-source, well-established tools. We used these tools offline to maintain data privacy and security. A limitation of our protocol is that, due to resource constraints, we were limited to spot-checks of alignments. However, in subsequent work using this dataset, we have observed differences in timing features extracted using word boundaries estimated from transcripts generated using different ASRs [27]. Further work, including manual verbatim and phonetic transcriptions, is required to explore the effects of different ASR tools on the quality and reliability of transcripts and to assess alignment accuracy and isolated vowels [91].

### Analysis Plan

#### Overview

We will use data collected using this protocol to assess within-participant variability in speech features within 1 day and 1 week and between recording devices and elicitation tasks. The analysis will include the features we have already extracted (Table 2) as well as suitable linguistic features (see examples in the study by Botelho et al [92]) extracted from the picture description tasks.

Our analyses will be in 3 stages. First, we will use test-retest scatter plots to visualize systematic versus random differences between pairs of recording sessions. Second, we will use linear mixed effect models [93,94] to estimate the within- and between-person variance. Each feature will be tested in a separate model. The models will include a participant random intercept and 2 dummy variables indicating whether the recording was made in the middle of the collection period (lunchtime for recordings over 1 day; Wednesday for recordings over 1 week) or later (evening or Friday). Third, from these models, we will calculate the ICC, the proportion of variance attributable to between-person differences (0=all variation is within-person and 1=all variation is between-person).

We will use linear mixed effect models to estimate differences in speech features over the day or week, using separate models for each feature. The models will include a participant random intercept and 2 dummy variables indicating whether the recording was made in the middle of the period (lunchtime for recordings over 1 day; Wednesday for recordings over 1 week) or later (evening or Friday).

We will additionally conduct a device-type sensitivity analysis, also using linear mixed effects models, to compare recordings from our benchmark condenser microphone with other devices we used, as in the study by Botelho et al [25]. This will reveal how within-speaker variability is captured in recordings by different mobile devices, which commonly use preprocessing and whose microphone specifications may vary. This analysis is needed to increase our understanding of the ability of mobile health tools to reliably capture changes in speech in research and clinical practice. We expect to complete this analysis and submit the follow-up paper later in 2025.

#### Data Utility

The core research question we set out to investigate with the data collected with this protocol in developing our protocol was within-individual speech variation within 1 day and 1 week, toward longitudinal assessments of health. However, the resulting data have broader utility in speech research and therefore represent value for funding. This is important to consider in study design, given the large resources needed to generate speech corpora.

We have begun using the data to benchmark different speech technologies (eg, ASR) and quantify associated variability in the feature extraction pipeline [27]. We have also demonstrated practice effects in repeated readings [95]. Further utility is gained from recording over multiple devices and using different elicitation methods, allowing us to assess variability in speech features according to these key methodological choices. It is vital to characterize such variation in speech over repeated speech samples to identify and develop reliable speech marker pipelines for clinical research and practice. Finally, we are also preparing to make the datasets accessible to other nonprofit researchers, enabling other investigations.

### Conclusions

In the speech-based health assessment literature, core methodological details and speaker characteristics are often underreported or the rationale for choices not explained. Underlying this, there is a need for more considered design of speech data curation. With this in mind, we have described a protocol for collecting nonpathological repeated speech samples. The core methodological aspects of this protocol cover design and reporting decisions that are relevant for researchers collecting longitudinal data for speech and language biomarker research. We encourage other researchers to adopt similar practices and consider the aspects we highlight in their own projects, thereby adding replicability and, ultimately, the translation of speech and language biomarkers into clinical research and practice.

## Appendix

Multimedia Appendix 1 Checklist of methodological aspects for consideration in protocol design and reporting.

Multimedia Appendix 2 Prerecording questionnaire completed by participants at the start of each recording session.

Multimedia Appendix 3 The day cohort reported recording times, median (IQR). Day 1 and day 2 were 8 to 11 weeks apart, on the same weekday.

Multimedia Appendix 4 Intervals between sessions, median (IQR) in minutes for day 1 and day 2 in the day study.

Multimedia Appendix 5 Distribution of recording times in the week study.

## Abbreviations

ALS: amyotrophic lateral sclerosis
ASR: automatic speech recognition
Cape-V: Consensus Auditory-Perceptual Evaluation of Voice
ComParE: Computational Paralinguistics Challenge Set
eGeMAPs: extended Geneva Minimalistic Acoustic Parameter Set
ICC: intraclass correlation
MFA: Montreal Forced Aligner
RADAR-MDD: Remote Assessment of Disease and Relapse—Major Depressive Disorder

## Data Availability

All code is available upon request from the corresponding author. Datasets will also be made available subject to participant consent and completion of a data use agreement.
