# Supplementary material for: Collection and Analysis of Repeated Speech Samples: Methodological Framework and Example Protocol
Source: JMIR Res Protoc. 2025 Jul 22;14:e69431. doi: 10.2196/69431 (PMC12326161; doi:10.2196/69431)
Supplement: Multimedia Appendix 1 [file resprot_v14i1e69431_app1.docx]

## Multimedia Appendix 1

Checklist of methodological aspects for consideration in protocol design and reporting

| Aspect | | Core considerations |
| --- | --- | --- |
|  |  |  |
| **Participants** | |  |
|  | Input and Feedback | Active involvement of patients and the public in clinical speech analytics development is critical to ensure technologies meet real-world needs. Patient and Public Involvement and Engagement (PPI-E) should be considered from the development stage of any speech collection project and can include the following aspects:   - project descriptions, and consent documents - recording device and set-up - privacy concerns and data retention for future research - acceptability of speech elicitation prompts - participant instructions - choice of speech measures - choice of clinical outcome and analysis |
|  | Eligibility criteria | Inclusion criteria and their implications for answering the research question in mind, including the presence of confounding factors, and recruitment feasibility:   - sociodemographic factors, e.g., gender, sex, age, education level, language, ethnicity - vocal tract and hearing disorders - respiratory conditions - mental health disorders - neurological disorders - medications prescribed / recently taken - lifestyle factors, e.g., smoking status and alcohol consumption |
|  | Recruitment | Recruiting a planned sample size within a defined time frame is a key bottleneck in speech research, particularly if a specific balance in, e.g., gender or age is sought. Key aspect to consider include:   - sociodemographic and socioeconomic biases - clinical vs general populations - whether partnerships with advocacy groups, clinical centers or related organizations are needed - Time limitations due to funder requirements |
| **Data Collection** | | Changes in speech are dictated by a range of speaker-specific factors and recording and analysis choices. It is important to collect and report information relating to these factors as they may relate to selection, information or confounding biases. |
|  | Metadata | The collection and reporting of participant characteristics that may be potential confounders balanced against ethical principles and regulatory requirements to avoid the unnecessary collection of sensitive participant data |
|  | Clinical assessments | How core clinical outcomes will be assessed. We recommend the use of validated scales and tests, where possible. Considerations include   - whether tests are clinician vs self-reported - time required to complete assessment and the associated participant burden |
|  | Recording devices | Effects of recording device, environment and time; these can all cause subtle changes in speech measurement. Aspects to consider and report include:   - mobile versus non-mobile devices - omni-directional or uni-directional microphones - ambient noise in the recording location - affordability and accessibility - device-specific signal processing - gain settings |
|  | Recording set-up and environment | Consistent conditions and device-speaker positioning are ideal and relevant factors include:   - recording device positioning requirements - ambient noise and room acoustics - participant comfort - whether participants stand or sit - office furniture features, e.g. adjustable seating - positioning of prompts/reading materials - remote versus in-lab or in-clinic data collection - collection of background noise for reporting of signal-to-noise ratio |
|  | Speech elicitation task | Choosing the optimal task to maximize the likelihood of identifying key correlates, clinical or otherwise. Factors to consider include:   - whether voice warm-up and familiarization is needed - practice effects and their implications for analysis - task order and risk of associated bias - task difficulty - task acceptability (established through PPI-E) - collection Procedure (some tasks, e.g., sustained phonation, may require more detailed instructions than others, this could the validity of the recorded sample) |
|  | Participant instructions | Decide strategies for instructing participants during sessions informed by PPI-E, adjusting as necessary during the study. Factors to consider include:   - how best to ensure reproducible positioning - participant mental and physical comfort throughout session - how to elicit natural speech - how to provide feedback and encouragement |
|  | Data Quality Log | Any incidents or participant behavior, or deviations from the protocol should be logged, where possible, to aid interpretation of the recordings and features subsequently extracted |
| **Data Processing** | | There are many ways in which we can digitize and process speech that can all affect the recorded signal and influence analysis. |
|  | Digitization | Digitization dictates the information captured and stored. Factors to consider and report include choice of   - sampling rate - bit rate - audio file format |
|  | Data Preparation | Resources required to prepare data for feature extraction may be considerable   - remove audio before and after each elicitation task - separate different tasks into individual files, where different elicitation tasks are captured in a single file perform any manual checks required, including when automated methods are used |
|  | Preprocessing | Application and reporting of the use of denoising, dereverberation, signal enhancement, speaker separation, and other similar audio processing tools; typically, these are not explicitly developed for clinical applications and may remove or alter health-related signals in the speech. Use of any such tool must be reported. |
|  | Feature selection | - whether the chosen features are measuring a speech construct related to the clinical outcome - whether interpretable/explainable features are needed |
|  | Feature extraction | Feature extraction methodology is a source of variability, including:   - choice of transcription tool (if used) and choice of offline or cloud-based use - choice of alignment tool (if used) - choice of feature extraction software and key settings - level of feature extraction (e.g., suprasegmental vs vowels) - criteria for and, identification and removal of outliers |
