# Supplementary material for: Collection and Analysis of Repeated Speech Samples: Methodological Framework and Example Protocol
Source: JMIR Res Protoc. 2025 Jul 22;14:e69431. doi: 10.2196/69431 (PMC12326161; doi:10.2196/69431)
Supplement: Multimedia Appendix 2 [file resprot_v14i1e69431_app2.docx]

| 1 | Are you experiencing any minor health issues today that may affect your voice? e.g., hay fever. | |
| --- | --- | --- |
|  | | Yes – please tell us here |
|  | | No |
|  | | Unsure – please tell us here |
| 2 | At what time did you wake up today? Please answer to the nearest 15 minutes. | |
| 3 | At what time did you get out of bed today? Please answer to the nearest 15 minutes. | |
| 4 | To the nearest hour, how many hours of sleep did you have last night? | |
| 5 | Which of the following best describes how you have used your voice so far today? | |
|  |  | Low Activity: I have spoken for less than one hour today / I haven’t spoken above conversational volume / I haven’t spoken in a group discussion, been teaching, or given a presentation or equivalent |
|  |  | Intermediate Activity: I have been talking intermittently to frequently today / I have raised my voice above conversational levels for short spells |
|  |  | High Activity: I have been talking for long spells today / I have been talking loudly and/or with an expressive voice / I have been teaching, have given presentations and/or performances |
| 6 | When did you last drink something? | |
|  |  | I had something to drink when I arrived at the recording session |
|  |  | Within the last hour |
|  |  | More than 1 hour ago |
|  |  | More than 2 hours ago |
|  |  | More than 3 hours ago |
| 7 | When did you last eat something? | |
|  |  | I had something to drink when I arrived at the recording session |
|  |  | Within the last hour |
|  |  | More than 1 hour ago |
|  |  | More than 2 hours ago |
|  |  | More than 3 hours ago |
| 8 | How are you? Select the image number from Pick-A-Mood that best describes how you feel at the moment. | |
| 9 | [on a separate screen] To follow up the picture you chose, which of these best describes how you are feeling at the moment? *This question is optional* | |
|  |  | neutral |
|  |  | excited-lively |
|  |  | cheerful-happy |
|  |  | tense-nervous |
|  |  | irritated-annoyed |
|  |  | sad-gloomy |
|  |  | bored-weary |
|  |  | calm-serene |
|  |  | relaxed-carefree |
