# Supplementary material for: Collection and Analysis of Repeated Speech Samples: Methodological Framework and Example Protocol
Source: JMIR Res Protoc. 2025 Jul 22;14:e69431. doi: 10.2196/69431 (PMC12326161; doi:10.2196/69431)
Supplement: Multimedia Appendix 3 [file resprot_v14i1e69431_app3.docx]

| Session | Time, Day 1 | Time, Day 2 |
| --- | --- | --- |
|  |  |  |
| **Morning (S1)** | | |
|  | 09:12 (08:43, 09:53) | 09:11 (08:33-09:53) |
| **Afternoon (S2)** | | |
|  | 14:05 (13:25-14:41) | 14:05 (13:07-14:42) |
| **Evening (S3)** | | |
|  | 18:04 (17:35-18:38) | 18:04 (17:30-18:41) |
