# Supplementary material for: Collection and Analysis of Repeated Speech Samples: Methodological Framework and Example Protocol
Source: JMIR Res Protoc. 2025 Jul 22;14:e69431. doi: 10.2196/69431 (PMC12326161; doi:10.2196/69431)
Supplement: Multimedia Appendix 4 [file resprot_v14i1e69431_app4.docx]

|  | Day 1 intervals | Day 2 intervals |
| --- | --- | --- |
|  |  |  |
| **S1-S2** | | |
|  | 291 (287, 292) | 293 (290, 298) |
| **S2-S3** | | |
|  | 240 (237, 241) | 240 (238, 241) |
| **S1-S3** | | |
|  | 529 (525, 533) | 534 (530, 538) |
