# Supplementary material for: Collection and Analysis of Repeated Speech Samples: Methodological Framework and Example Protocol
Source: JMIR Res Protoc. 2025 Jul 22;14:e69431. doi: 10.2196/69431 (PMC12326161; doi:10.2196/69431)
Supplement: Multimedia Appendix 5 [file resprot_v14i1e69431_app5.docx]

| Recording interval | Number of participants |
| --- | --- |
|  |  |
| 09:00-10:00 | 1 |
| 10:00-11:00 | 5 |
| 11:00-12:00 | 2 |
| 12:00-13:00 | 5 |
| 13:00-14:00 | 2 |
| 14:00-15:00 | 2 |
| 15:00-16:00 | 3 |
| 16:00-17:00 | 4 |
| 17:00-18:00 | 1 |

## 
